# GlyPerA™ effectively shields airway epithelia from SARS-CoV-2 infection and inflammatory events

**DOI:** 10.1186/s12931-023-02397-3

**Published:** 2023-03-22

**Authors:** Viktoria Zaderer, Stefanie Dichtl, Wilfried Posch, Ivane Abiatari, Günther K. Bonn, Thomas Jakschitz, Lukas A. Huber, Teymuras V. Kurzchalia, Doris Wilflingseder

**Affiliations:** 1 Institute of Hygiene and Medical Microbiology, Medical University of Innsbruck, Schöpfstrasse 41/R311, 6020 Innsbruck, Austria; 2 School of Natural Sciences and Medicine Tbilisi, Ilia State University, Tbilisi, Georgia; 3 Austrian Drug Screening Institute (ADSI), Innsbruck, Austria; 4 Institute of Cell Biology, Biocenter Medical University of Innsbruck, Innsbruck, Austria; 5 Dzala LLC, 3 Gotua Str., 0160 Tbilisi, Georgia

**Keywords:** SARS-CoV-2, Prophylaxis, Variants of concern, Antiviral, Transmission

## Abstract

New SARS-CoV-2 variants of concern (VOCs) and waning immunity illustrate that quick and easy-to-use agents are needed to prevent infection. To protect from viral transmission and subsequent inflammatory reactions, we applied GlyperA™, a novel antimicrobial formulation that can be used as mouth gargling solution or as nasal spray, to highly differentiated human airway epithelia prior infection with Omicron VOCs BA.1 and BA.2. This formulation fully protected polarized human epithelium cultured in air–liquid interphase (ALI) from SARS-CoV-2-mediated tissue destruction and infection upon single application up to two days post infection. Moreover, inflammatory reactions induced by the Omicron VOCs were significantly lowered in tissue equivalents either pre-treated with the GlyperA™ solution, or even when added simultaneously. Thus, the GlyperA™ formulation significantly shielded epithelial integrity, successfully blocked infection with Omicron and release of viral particles, and decreased intracellular complement C3 activation within human airway epithelial cell cultures. Crucially, our in vitro data imply that GlyperA™ may be a simple tool to prevent from SARS-CoV-2 infection independent on the circulating variant via both, mouth and nose.

**Supplementary Information:**

The online version contains supplementary material available at 10.1186/s12931-023-02397-3.

## Introduction

Novel SARS-CoV-2 variants of concern (VOC) rapidly appear and the Omicron variants BA.1 (B.1.1.529.1) and BA.2 (B.1.1.529.2) exerted a higher contagiousness compared to earlier variants. Moreover, due to multiple mutations within the receptor-binding domain (RBD), such continuously emerging VOCs are potentially less susceptible against currently used vaccines [1]. Thus, additional, broadly available and easy-to-use preventive measures and prophylactic treatments are needed to prevent SARS-CoV-2 infections—in particular also in vulnerable groups, such as immunocompromised individuals, where vaccinations are less effective [2].

Posch et al. recently illustrated that complement C3 is intracellularly massively mobilized in human airway epithelial (HAE) tissue cultures after SARS-CoV-2 infection independent on the VOC used and this was associated with high anaphylatoxin and pro-inflammatory cytokine release, high viral loads and excessive tissue destruction [3, 4]. These virus-mediated effects could be efficiently blocked by antagonizing the anaphylatoxin receptor C5aR at basolateral sites of epithelial tissues, but also by applying the oral mouth spray ColdZyme® to highly differentiated primary nasal and bronchial epithelial 3D tissue models prior infection with SARS-CoV-2 wildtype virus [3–5]. Thus, we, here, tested the efficacy of another antimicrobial solution, GlyPerA™ that can be used as mouth gargling solution or as nasal spray against the highly contagious Omicron variants.

GlyPerA™ is a formulation consisting of active compounds hydrogen peroxide (H_2_O_2_) and glycine. Hydrogen peroxide, H_2_O_2_, is an established antiseptic that destroys bacteria, viruses and fungi. Since many decades it is used: for disinfection of material surfaces (e.g. of medical instruments or devices), for sterilization of water and raw materials for food production (e.g. diary products), as antimicrobial agent for vegetable/fruit skin treatment, for disinfection of wounds and as antimicrobial agent for sanitation of oral or outer ear cavities. Although being a GRAS (Generally regarded as safe) substance for the applications mentioned above, amounts used are strictly regulated because H_2_O_2_ in high concentrations and frequent or long-term application damages cells. The major target of hydrogen peroxide in the eukaryotic cell is mitochondria. It was discovered that a naturally occurring small carboxylic acid, glycolic acid, can ameliorate harmful effects of paraquat, a strong oxidant that produces Reactive Oxygen Species (including superoxide and hydrogen peroxide), on mitochondria [6]. More recently it was shown that this endogenous compound acts by entering serine/glycine metabolism. In this way, conversion of glycolate into glycine boosts cellular Glutathione-defence system and neutralizes damage induced by H_2_O_2_ treatment [7]. Thus, GlyPerA™ exploits the principle: high concentration of hydrogen peroxide destroys microbes (among them viruses) outside of cells, whereas glycine neutralizes adverse effects of the former within cells. This makes possible to use GlyPerA™ for longer periods of time and more frequently then pure H_2_O_2_.

While tissue destruction with concomitant innate immune–complement C3—activation and high release of viruses into the basolateral subnatant was detected upon infection of normal human bronchial epithelial (NHBE) cells with Omicron VOCs BA.1 and BA.2, tissue integrity was completely restored and local complement C3 production and virus release significantly down-modulated by single application of the GlyPerA™ solution. Since this solution cannot only be used orally, but also intranasally, our results point towards an easy-to-use prophylactic treatment using GlyPerA™ in order to prevent infection with highly contagious novel SARS-CoV-2 VOCs.

## Materials and methods

### Ethics statement

Written informed consent was obtained from all donors of leftover nasopharyngeal/ oropharyngeal specimen and EDTA blood by the participating clinics. The Ethics Committee of the Medical University of Innsbruck (a copy is attached to the proposal, ECS1166/2020) approved the use of anonymized leftover specimens of COVID-19 patients for scientific purposes.

### Cell culture of tissue models and GlyPerA™ treatment

#### Human Airway Epithelia (HAE)

Normal human bronchial epithelial (NHBE, Lonza, cat# CC-2540S, Germany) are available in our laboratory and routinely cultured in air liquid interface (ALI) as described [8, 9]. Briefly, cells were cultured in a T75 flask for 2–4 days until they reached 80% confluency. The cells were trypsinized and seeded onto GrowDexT (UPM)-coated 0.33 cm^2^ porous (0.4 µm) polyester membrane inserts with a seeding density of 1 × 10^5^ cells per Transwell (Costar, Corning, New York, NY, USA). The cells were grown to near confluency in submerged culture for 2–3 days in specific epithelial cell growth medium according to the manufacturer´s instructions (PneumaCult™-Ex Plus Medium, Stemcell, cat# 05040, Germany). Cultures were maintained in a humidified atmosphere with 5% CO_2_ at 37 °C and then transferred to ALI culture in PneumaCult™-ALI medium (Stemcell, cat# 05001, Germany) for another 30 days until fully differentiated.

25 µl GlyPerA™ were apically applied pure or diluted (1/100) 5 min prior infection using BA.1 or BA.2 (GlyperA pure PRE Omicron; GlyperA dil. PRE Omicron). In addition, the diluted solution was added simultaneously (GlyPerA dil. SIM Omicron) or 45 min post infection (GlyPerA dil. POST Omicron) to the apical side of the fully differentiated epithelia prior to infection using BA.1 or BA.2 at an MOI 0.005. The apical application by pipetting was carefully performed to not mechanically disrupt the epithelial surface.

### Viruses

Clinical specimens for SARS-CoV-2 Omicron BA.1 (B.1.1.529 BA.1) and BA.2 (B.1.1.529 BA.2) from COVID-19 positive swabs, sequenced by the Austrian Agency for Health and Food Safety, Vienna, Austria were propagated in Vero cells and subsequently used to infect cells.

#### Vero cells

VeroE6/TMPRSS2/ACE2 is an engineered VeroE6 cell line expressing high levels of TMPRSS2 and ACE2 and highly susceptible to SARS-CoV-2 infection. This cell line was used to expand characterized BA.1 and BA.2 viruses from patient isolates. The cell line was obtained via the CFAR (NIBSC) and is described in [10].

### TEER measurement

TEER values were measured using EVOM volt-ohm-meter with STX-2 chopstick electrodes (World Precision Instruments, Stevenage, UK). Measurements on cells in ALI culture were taken immediately before the medium was exchanged. For measurements, 0.1 ml and 0.7 ml of medium were added to the apical and basolateral chambers, respectively. Cells were allowed to equilibrate before TEER was measured. TEER values reported were corrected for the resistance and surface area of the Transwell filters.

### Real-time RT-PCR for absolute quantification of SARS-CoV-2

SARS-CoV-2 RNA was extracted using FavorPrep Viral RNA Mini Kit, according to manufacturer’s instructions (Favorgen Europe, cat# FAVRE 96004, Austria). Sequences specific to 2 distinct regions of the Nucleocapsid (N) gene, N1 and N2, and for the detection of a human housekeeping gene, Ribonuclease P, were used. Single target assays of all 3 targets were performed in combination with the Luna Universal Probe One-Step RT-qPCR Kit (New England Biolabs, cat# E3006, Germany). For absolute quantification using the standard curve method, SARS-CoV-2 RNA was obtained as a PCR standard control from the National Institute for Biological Standards and Control, UK. All runs were performed on a Bio-Rad CFX 96 instrument and analyzed by the Bio-Rad CFX Maestro 1.1 software (Bio-Rad, Germany).

### Staining and high content screening (HCS)

To visualize SARS-CoV-2 infection in monolayers and 3D tissue models, cells were infected with clinical specimen of SARS-CoV-2 BA.1 and BA.2 and analyzed for characteristic markers in infection experiments on day 3 post infection (3 dpi). After SARS-CoV-2 exposure, 3D cell cultures were fixed with 4% paraformaldehyde. Intracellular staining was performed using 1 × Intracellular Staining Permeabilization Wash Buffer (10X; BioLegend, San Diego, CA, USA). Nuclei were detected using Hoechst 33342 (Cell Signaling Technologies, cat# 4082, Netherlands), complement C3 using a C3-FITC (Agilent Technologies, cat# F020102-2, Austria) and cilia using an acetylated Tubulin-Alexa647 antibody (Abcam, cat# ab218591, UK). Intracellular SARS-CoV-2 was detected using Alexa594-labeled SARS-CoV-2 antibodies against S1 and N (both Sino Biological, Beijing, China). The Alexa594-labeling kit was purchased from Abcam, Cambridge, UK. After staining, 3D cultures were mounted in Mowiol. To study these complex models using primary cell cultured in 3D and to generate detailed phenotypic fingerprints for deeper biological insights in a high throughput manner, the Operetta CLS System (PerkinElmer, Waltham, MA, USA) was applied. For analyzing nuclei counts, spot analyses for SARS-CoV-2 particles and C3 area, Harmony™ Software was used and analyses performed in more than 1500 cells per condition.

### Statistical analysis

Statistical analysis of differences in infection levels, TEER values, cell numbers, virus spots or C3 areas was performed utilising the GraphPad prism software and using OneWay ANOVA with Tukey´s post test.

## Results

### GlyPerA™ mouth spray maintains epithelial integrity upon SARS-CoV-2 infection of NHBE cultures

**Fig. 1:**
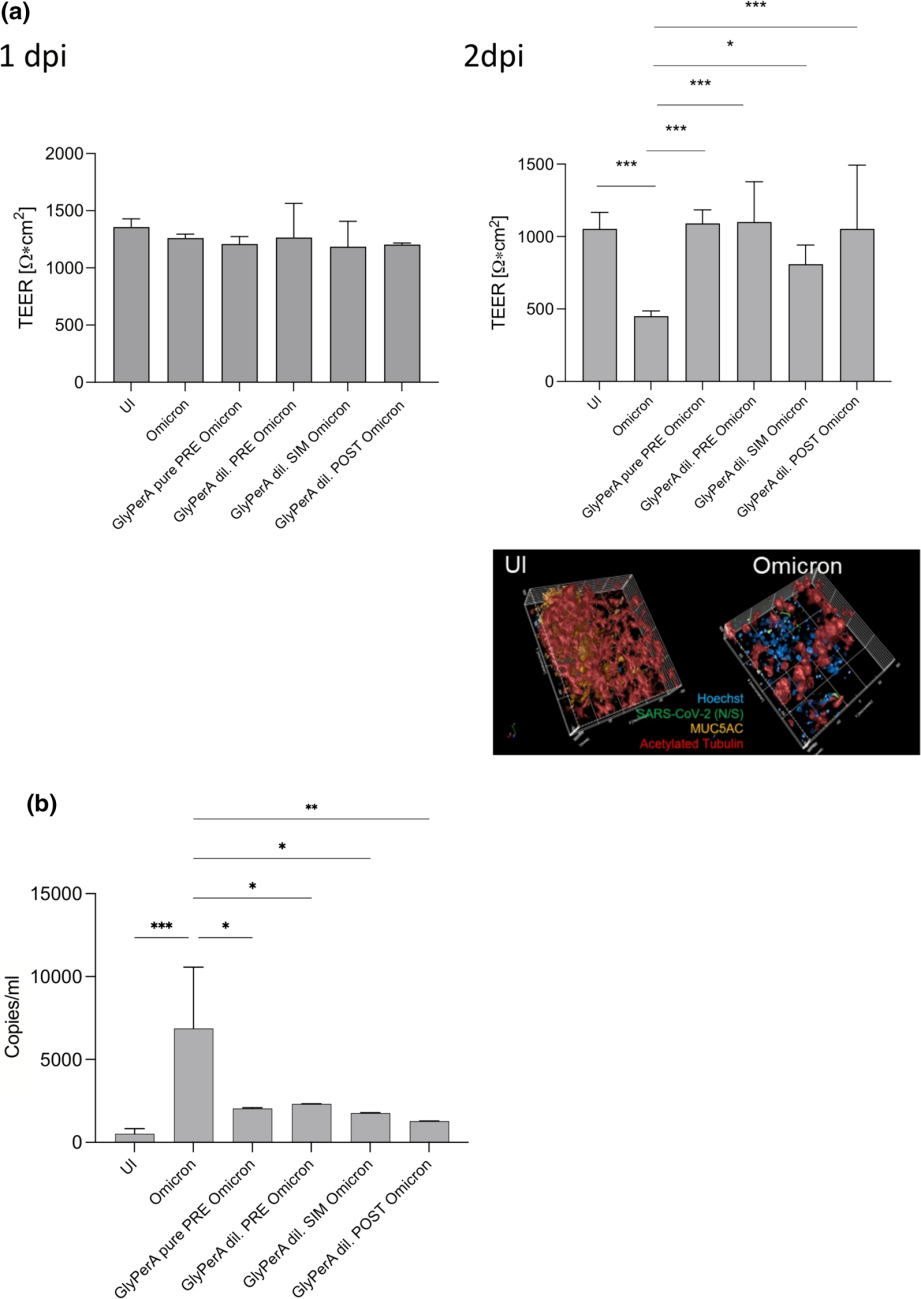
Disruption of epithelial integrity by Omicron BA.1 or BA.2 can be avoided by GlyPerA™ treatment. **a** Pseudostratified epithelia were infected by apical addition of SARS-CoV-2 BA.1 and BA.2 with or without GlyPerA™ treatment and incubated for 48 h. GlyPerA™ solution was added pure or diluted (1/100) 5 min prior infection (GlyPerA pure PRE Omicron, GlyPerA dil. PRE Omicron), and the diluted solution in addition simultaneous with (GlyPerA dil. SIM Omicron) or 45 min post (GlyPerA dil. POST Omicron) infection. TEER was measured on 1 dpi (left) and 2 dpi (right) using a EVOM volt-ohm-meter. TEER in Ω/cm^2^ was determined for all conditions (UI, Omicron, GlyPerA pure PRE Omicron, GlyPerA dil. PRE Omicron, GlyPerA dil. SIM Omicron, GlyPerA dil. POST Omicron) and plotted on a bar graph. Bars represent the mean + SD from 3 independent pseudostratified epithelia measured in triplicates. Statistical significance was calculated using One-way ANOVA with Tukey’s multiple comparisons test. To further validate the drop in TEER, immunofluorescence analyses were performed to stain for the tissue integrity. For this, 2 dpi tissue models were stained using Hoechst (nuclei, blue), acetylated tubulin (cilia, red), MUC5AC (mucus-producing cells, orange) and virus (SARS-CoV-2, green). **b** Viral RNA was measured from UI, Omicron, GlyPerA pure PRE Omicron, GlyPerA dil. PRE Omicron, GlyPerA dil. SIM Omicron, GlyPerA dil. POST Omicron cultures of pseudostratified epithelia on 3 dpi. The experiment was repeated at least 3 times and statistically significantl differences were determined by one-way ANOVA with Tukey´s multiple comparisons test. All values are means ± SD. In **a** and **b** *P < 0.05, **P < 0.01, ***P < 0.001

To monitor, if single administration of the GlyPerA™ solution, which can be used as mouth- or nose spray, protects respiratory tissues from SARS-CoV-2-mediated destruction over time, NHBE cultures were kept in culture for 2 days following treatment and infection. The solution was either added pure (GlyPerA pure) or diluted 1/100 (GlyPerA dil.) 5 min prior infection with BA.1 or BA.2 at an MOI 0.005. The diluted solution was further added simultaneous to infection (GlyPerA dil. SIM Omicron) or 45 min post infection (GlyPerA dil. POST Omicron) with Omicron. Analysis of transepithelial electrical resistance (TEER; Fig. 1a), an indicator for tissue integrity, was performed on 1 dpi and 2 dpi. These analyses revealed that upon Omicron infection, TEER values slightly dropped 1 dpi (left) and significantly decreased 2 dpi (right) compared to GlyPerA-treated UI controls (UI; Fig. 1a). This drop in TEER was also consistent with tissue destruction on 2 dpi analyzed by immunofluorescence analyses (Fig. 1a, lower right; Additional file 1: Fig. S1, right) and LDH from basolateral supernatants as cytotoxicity measure (Additional file 1: Fig. S1, left). While in GlyPerA-treated UI controls, an intact ciliated layer (red) with mucus-producing goblet cells (orange) was observed, Omicron-infected tissues illustrated porous, infected (green) tissue structures (Fig. 1a, lower right). Nuclei were stained using Hoechst (Fig. 1a, lower right, blue). Moreover, occludin staining revealed fully intact tight junctions in GlyPerA-treated UI controls, while these were lost in Omicron-infected tissue models on 2 dpi (Additional file 1: Fig. S1, occludin in blue, right). GlyPerA treatment significantly rescued epithelial integrity on 2 dpi (right) independent on the concentration of GlyPerA (pure vs. diluted) and time of administration (pre, sim, post) in infected cultures (Fig. 1a). TEER values of GlyPerA-treated and Omicron-infected tissues were in the range of the mock-treated, uninfected control (Fig. 1a). Thus, tissue integrity was greatly rescued in infection experiments on both days analyzed post infection, if GlyPerA™ solution was administered prior, simultaneous to and post SARS-CoV-2 exposure only once.

### GlyPer™ addition significantly lowers SARS-CoV-2 viral loads compared to infected controls independent on the time of administration

In accordance to TEER, absolute quantification of viral load from 3 dpi subnatants of differently treated cells revealed protection from infection by applying the GlyPer™ solution (Fig. 1b). The protection from viral infection was independent on whether the GlyPer™ formulation was added shortly prior infection (GlyPerA pure PRE and GlyPerA dil. PRE Omicron), simultaneously (GlyPerA dil. SIM Omicron) or 45 min post infection with BA.1 or BA.2 (GlyPerA dil. POST Omicron) (Fig. 1b). Viral copy numbers in GlyPerA-treated and infected subnatants were slightly higher than background levels of uninfected (UI) and significantly lower compared to Omicron-infected (Omicron) cultures (Fig. 1b). Here, we demonstrated that single application of GlyPerA™ solution blocked SARS-CoV-2 infection of HAE cultures by significantly reducing virus release independent on the time of administration.

### GlyPerA™ protects from SARS-CoV-2 infection and intracellular (IC) C3 activation in primary HAE cells

Lastly, we monitored primary NHBE cell infection and inflammation after applying SARS-CoV-2 variants of concern (VOCs) Omicron BA.1 and BA.2 in absence and presence of GlyPerA™ solution by imaging analyses. In these experiments, a multiplicity of infection (MOI) of 0.005 was used to determine morphogenesis, inflammation (IC C3 activation) and cytopathic effects of SARS-CoV-2 infection in human airway epithelial cells.

**Fig. 2:**
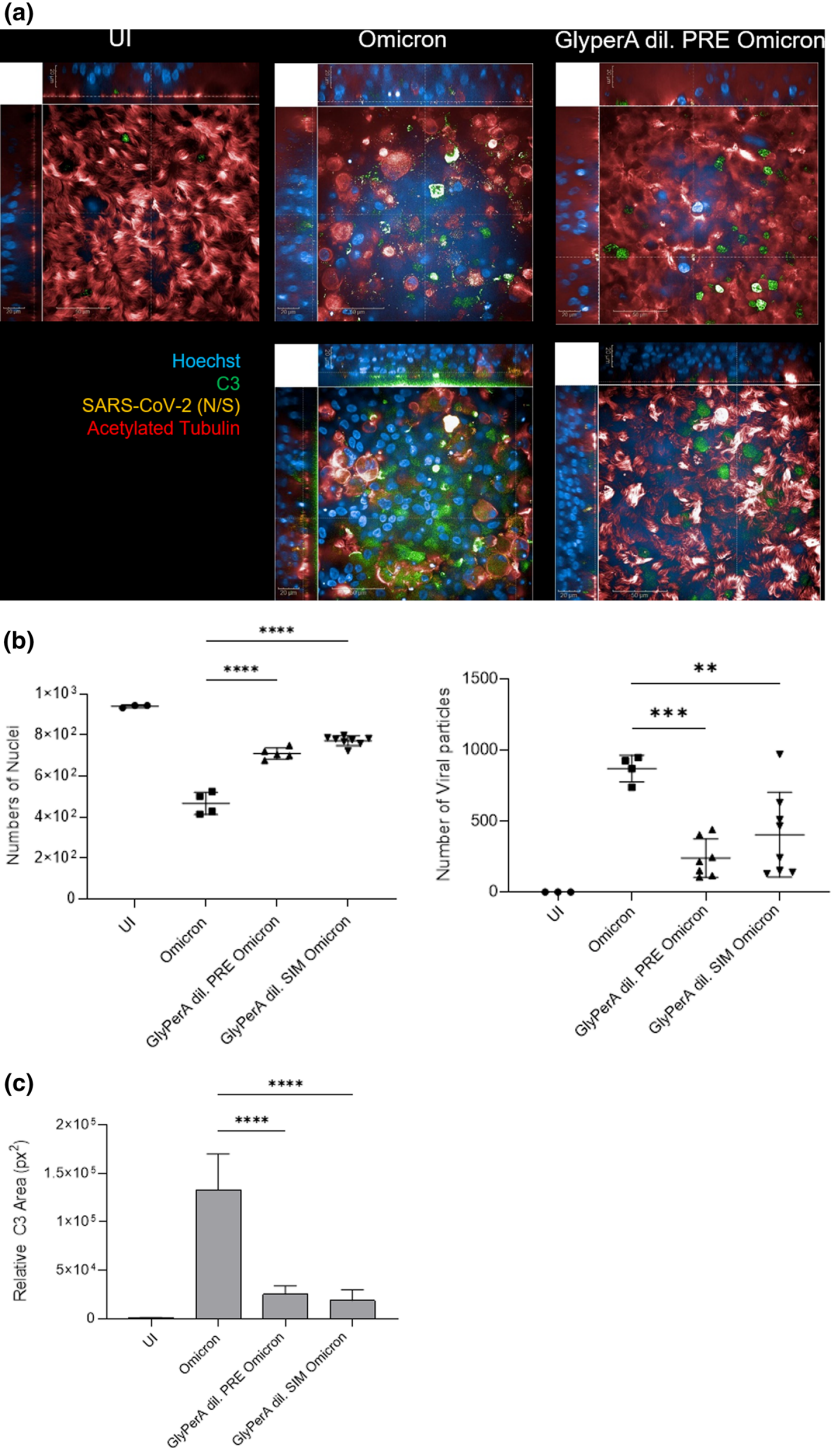
GlyPerA™ shields primary HAE cells from SARS-CoV-2 Omicron BA.1 and BA.2 infection and innate immune activation. Visualization of virus binding (SARS-CoV-2 S1/N, orange) and complement (C3-FITC, green) in SARS-CoV-2 infected 3D pseudostratified epithelia. Pseudostratified epithelia were apically treated with GlyPerA™ prior exposure to SARS-CoV-2. On 3 dpi, filters were fixed, stained for höchst (blue), SARS-CoV-2 S1/N (orange), complement C3 (green) and acetylated tubulin (red) and then analysed by HCS. **a** XYZ-stacks of uninfected (UI, panel 1), BA.2-infected (panel 2), GlyPerA™-pre-treated and BA.2-infected (panel 3) HAE cultures were analyzed using the Operetta CLS HCS and the 63XWATER objective. Cells were stained using C3-FITC (green) as indicator for innate immune activation, SARS-CoV-2-S1/N-Alexa594 (orange) for virus detection, höchst for imaging nuclei (blue) and acetylated tubulin for staining cilia (red). High IC C3 mobilization was monitored in BA.2-infected cultures, while no virus and low C3 signals were detected in UI (panel 1) and GlyPerA™/BA.2- (panel 3) cultures. Scale bars represent 50 µm (XY) and 20 µm (YZ, XZ), respectively. Three independent experiments were performed. **b** Numbers of nuclei per condition (UI, Omicron, GlyPerA dil. PRE Omicron, GlyPerA dil. SIM Omicron), viral signals and **c** areas of C3 production were absolutely quantified from at least three different areas using the Harmony™ 4.9 software. Statistical significances were analyzed with GraphPad Prism software using One-way ANOVA and Tukey’s post test. In **b** and **c** **P < 0.01, ***P < 0.001, ****P < 0.0001

GlyPerA™ solution was carefully added onto the apical side of fully differentiated, pseudostratified epithelia cultured at air–liquid interphase as described by us [3–5, 8, 9] to realistically mimic the distribution within the oral or nasal cavity. Epithelia were incubated for 5 min with diluted GlyPerA™ solution prior applying clinical isolates derived from BA.1- or BA.2-infected individuals or the solution was added simultaneously with the viral preparations. The clinical specimen were anonymized before use and the study has been approved by the ethics committee of the Medical University of Innsbruck (approval no. ECS1166/2020). Uninfected tissues treated with GlyPerA™ solution alone (UI) served as control in all imaging experiments. After 3 days post infection (3 dpi), tissue models were fixed and stained for immunofluorescence (IF) analyses using Alexa594-labeled antibodies against the SARS-CoV-2 spike 1 (S1) and nucleocapsid (N) proteins (Fig. 2a and Additional file 2: Videos S1a and Additional file 3: S1b, orange, SARS-CoV-2 (N/S)) to detect virus, Hoechst for nuclei (Fig. 2a and Additional file 2: Videos S1a and Additional file 3: Video S1b, blue), acetylated tubulin for cilia (Fig. 2a and Additional file 2: Video S1a and Additional file 3: Video S1b, red), and complement component C3 as marker for innate immune activation of NHBE cells (Fig. 2a and Videos S1a and Additional file 3: Video S1b, green).

We recently found that infection in primary airway epithelial cells was escorted by massive intracellular (IC) C3 mobilization and secretion of the anaphylatoxin C3a from HAE cells [4]. Thus, we used IC C3 as marker for innate immune activation during infection of NHBE cells with BA.1 and BA.2 in absence and presence of GlyPerA™.

Z-stack analyses of Omicron (BA.1 or BA.2)-infected NHBE cells treated with GlyPerA™ revealed an intact, pseudostratified epithelium with no detectable virus or virus-infected cells (Fig. 2a, right panels, and Video S1b). In contrast, epithelia infected with Omicron BA.1 or BA.2 illustrated extensive destruction of epithelia with virus-infected, inflamed cells (Fig. 2a, middle panels, and Video S1a, green, orange). GlyPerA-mock treated UI cells served as controls for intact epithelia; these showed the most intact cilia layer as well as the lowest IC C3 activation (Fig. 2a, left panel).

When absolutely quantifying nuclei in more than 1500 cells per condition, we found that Omicron infection significantly decreased the nuclei count compared to UI on 3 dpi (Fig. 2b, left). GlyPerA-treatment prior to or simultaneous with BA.1 or BA.2 significantly rescued cell numbers compared to infected cultures, but were decreased in comparison to UI (Fig. 2b, left). Moreover, GlyPerA treatment significantly rescued from infection compared to infected tissues independent on the time of addition (pre, sim), when viral particles were counted (Fig. 2b, right). These analyses are in accordance to absolutely quantified viral copy numbers using RT-PCR (Fig. 1b).

Lastly, Omicron infection was associated with concomitant activation of IC C3 (green) (Fig. 2a, middle panels, and Additional file 2: Video S1a), while the IC C3 mobilization was significantly decreased, when tissue samples were pretreated with GlyPerA solution (Fig. 2a, right panels, Fig. 2c, Additional file 3: Video S1b) or when the solution was added simultaneously (Fig. 2c).

Upon quantifying C3 signals from more than 1000 cells/condition in UI, Omicron- and GlyPerA/Omicron-exposed, pseudostratified respiratory epithelia, we found highly significant differences in percentages of C3^+^ areas (Fig. 2c) These analyses demonstrate that infection going along with tissue destruction and intracellular C3 mobilization on 3 dpi induced in NHBE cultures upon SARS-CoV-2 interactions can be avoided by pre-treatment with or simultaneous addition of GlyPerA™ solution to epithelia.

## Discussion

Here, we found that GlyPerA™ treatment was sufficient to block SARS-CoV-2 VOCs, Omicron BA.1 and BA.2, from infecting highly differentiated, mucus-producing and ciliated primary human bronchial airway epithelial tissue cultures (standardized in our laboratory as described in [3–5, 8, 9] and depicted in Additional file 1: Fig. S2). GlyPerA™ solution consists of two active compounds, hydrogen peroxide (H_2_O_2_) and glycine. This novel composition of H_2_O_2_ and glycine protects cells, tissues, and organs against the destructive actions of hydrogen peroxide-induced oxidative stress, while still allowing its antimicrobial and signaling activities [7]. GlyPerA™ solution is a GRAS substance and due to the H_2_O_2_ content, it acts as an efficient antimicrobial also against viruses outside of cells, while glycine neutralizes adverse effects of hydrogen peroxide within cells. We found that addition of glycine to hydrogen peroxide significantly improved cell survival of a human pancreatic cell line over 24 h (not shown). Since cell lines grown in 2D are more sensitive to applied drugs compared to 3D models [11], exhibiting more similar features to in vivo systems, GlyPerA™ solution will even have less impact on the survival of the cells in our ALI models. This was also highlighted in 3D imaging analyses of GlyPerA ™-treated, uninfected cultures that fully conserved their tissue structures (Fig. 2a, UI) as well as LDH cytotoxicity analyses (Additional file 1: Fig. S1). We found a significantly higher LDH activity in SARS-CoV-2-infected versus D-PBS- or GlyPerA™-treated uninfected or GlyPerA™-treated infected cultures. This highly effective mode of action and maintainance of tissue archidecture makes GlyPerA™ solution an easy-to-use product locally effective at mucosal sites due to its use as gargling solution or nasal spray. Since nasal and oral epithelium are portals for initial infection and transmission, and novel SARS-CoV-2 Omicron VOCs are highly contagious and might contain mutations allowing them to evade immunity obtained through vaccines or prior SARS-CoV-2 infection [12, 13], it is becoming clear that we need alternative and broadly effective strategies to avoid SARS-CoV-2 infection and transmission. We recently found that an antiviral mouth spray completely blocked infection with SARS-CoV-2 wildtype and Omicron variants BA.1, BA.4/0.5 and concomitant virus-induced tissue damage and local complement activation as indicator for innate immune activation [3, 5]. The SARS-CoV-2 variants did not differ in their susceptibility to the antiviral mouth spray ColdZyme® [3, 5], thus suggesting that also the GlyPerA™ solution is effective against upcoming SARS-CoV-2 VOCs like BA.4, BA.5, or XBB1.5. Aggravating of injury by local complement activation in the airway epithelium was shown for infection with SARS-CoV-2 during the first COVID-19 wave [14, 15]. Moreover, increased anaphylatoxin levels (C3a, C5a) in plasma and lung homogenates have been implicated in the pathogenesis of various lung conditions including cystic fibrosis and idiopathic pulmonary fibrosis [16, 17]. High anaphylatoxin levels were found to down-modulate regulators of complement activation such as CD55 and CD46 and changes in injured human airway epithelial markers [16]. As illustrated in our highly differentiated, pseudostratified 3D models, high levels of C3a were secreted from the airway epithelium upon interaction with SARS-CoV-2 [4]. Thus, we here analyzed not only effects of GlyPerA™ in terms of tissue integrity by measuring transepithelial electrical resistance, but also viral loads of infected tissues in absence and presence of the antimicrobial solution as well as intracellular C3 production. We applied pure or diluted GlyPerA™ solution to highly differentiated normal human bronchial epithelial cells of the upper respiratory tract grown in air–liquid interphase before, simultaneous with or after infection with SARS-CoV-2 Omicron variants, and found an effective virus inactivation by distributing the compound in a single administration independent on the time of application and on the concentration. Virus inactivation due to treatment of HAE tissue models with GlyPerA™ was concomitant with down-modulation of local complement production and maintainance of tissue integrity as also observed with the antiviral mouth spray [3, 5]. GlyPerA™-mediated antiviral and simultaneously cell-protecting effects observed in our model are due to the combination of H_2_O_2_ as antimicrobial or signaling agent at high concentrations and glycine as counteracter of hydrogen peroxide-induced cellular toxicity [7]. Normally, oxidative respiration of a cell in a living organism, especially mitochondrial respiration, results in generation of reactive oxygen species (ROS) and conversion to hydrogen peroxide production [18, 19]. H_2_O_2_ together with excessive iron produce the extremely aggressive hydroxyl radical that interacts with any chemical bond in vicinity [20, 21]. Moreover, SARS-CoV-2 E protein was postulated to attach to haem from attacked hemoglobin and phagocytes, thereby being able to synthesize oxygen and water into superoxide anions, hydrogen peroxide and hydroxyl radicals [22]. Consequently, glycine comes into play by neutralizing both before entry of cells, by simultaneously preserving the antiviral actions of GlyPerA™ solution. We earlier showed that excessive levels of C3a were produced upon SARS-CoV-2 infection in respiratory tissues—thus, intrinsic C3 generation is a good indicator for successive anaphylatoxin production at sites of infection [3–5]. Despite the milder pathogenesis described with Omicron compared to SARS-CoV-2 earlier variants [23], we here also found intrinsic complement C3 generation at infection sites within pseudostratified epithelia concomitant with tissue destruction on day 3 post infection and virus release. These effects were antagonized in primary human airway epithelia by single of epithelia with GlyPerA™ that was either pre-incubated, added simultaneously or also in experiments, where the solution was added post infection. Independent on the time of addition, epithelia exerted a high transepithelial electrical resistance, a marker of epithelial integrity, and also significantly lower intracellular C3 production and virus release, thus indicating that GlyPerA™ is effective in protecting not only from SARS-CoV-2-mediated infection, but also from tissue destruction and local inflammatory reactions. The use of GlyPerA™ as gargling solution and for nasal administration renders this device a simple and promising approach to protect from viral transmission events and in a secondary reaction, also from inflammatory and tissue-destructive actions.

Although results from our in vitro HAE models are not adaptable directly into in vivo efficacy, they open up possibilities to apply GlyPerA™ in the prevention of contagious SARS-CoV-2 VOC transmission and spread.

## Supplementary Information

**Additional file 1: Fig S1. **GlyPerA treatment of 3D human respiratory tissues protects from SARS-CoV-2-mediated cytotoxicity. (left) Cytotoxicity was analyzed using the Cytotoxicity Detection Kit (LDH) from Roche according to the manufacturer´s instructions (Merck, cat# 1164493001, Austria). This kit is based on measuring LDH activity released from damaged cells. Treatment of 3D human respiratory tissue models with GlyPerA at a dilution 1/100 (UI/GlyPerA dil.) did not harm the cells compared to D-PBS-treated control cells. In contrast, SARS-CoV-2 (Omicron)-infected cells showed a significantly higher LDH release, which was significantly down-modulated when cells were either pre-treated with GlyPerA-solution prior infection or when the substance was added simultaneously (GlyPerA dil. PRE Omicron, GlyPerA dil. SIM Omicron). This indicates a significant protection of the tissues from infection by the compound independent on the time of application. *P < 0.05 (right) An occludin staining revealed that GlyPerA-treatment of NHBE respiratory cultures did not have any effect on the tight junctions of epithelia, while in Omicron-infected cultures cells were destroyed. Occludin in blue, scale bars 50 µm. **Fig S2.** Characterization of ciliated and mucus-producing cells. Ciliated and mucus-producing cells were characterized in NHBE cells at ALI on 1 dpi with SARS-CoV-2 (red). Ciliated cells were stained using acetylated tubulin (green) and goblet cells using MUC5A (orange). The upper picture illustrates all stainings, while in the lower picture the Höchst signal was switched off to see the distribution of ciliated and goblet cells.

**Additional file 2: Video S1.** 3D illustration of Omicron- and GlyPerA-pre-incubated, Omicron-infected NHBE cells on 2 dpi. Ciliated cells were stained using acetylated tubulin (red), local inflammation of human airway epithelial cells using complement C3 (green), nuclei using Hoechst (blue) and virus is depicted in orange (SARS-CoV-2 (N/S), orange).

**Additional file 3: Video S1.** 3D illustration of Omicron- and GlyPerA-pre-incubated, Omicron-infected NHBE cells on 2 dpi. Ciliated cells were stained using acetylated tubulin (red), local inflammation of human airway epithelial cells using complement C3 (green), nuclei using Hoechst (blue) and virus is depicted in orange (SARS-CoV-2 (N/S), orange).

## Data Availability

All data generated or analysed during this study are included in this article.
