# Supplementary figures and images for: GlyPerA™ effectively shields airway epithelia from SARS-CoV-2 infection and inflammatory events
Source: Respir Res. 2023 Mar 22;24:88. doi: 10.1186/s12931-023-02397-3 (PMC10032620; doi:10.1186/s12931-023-02397-3)

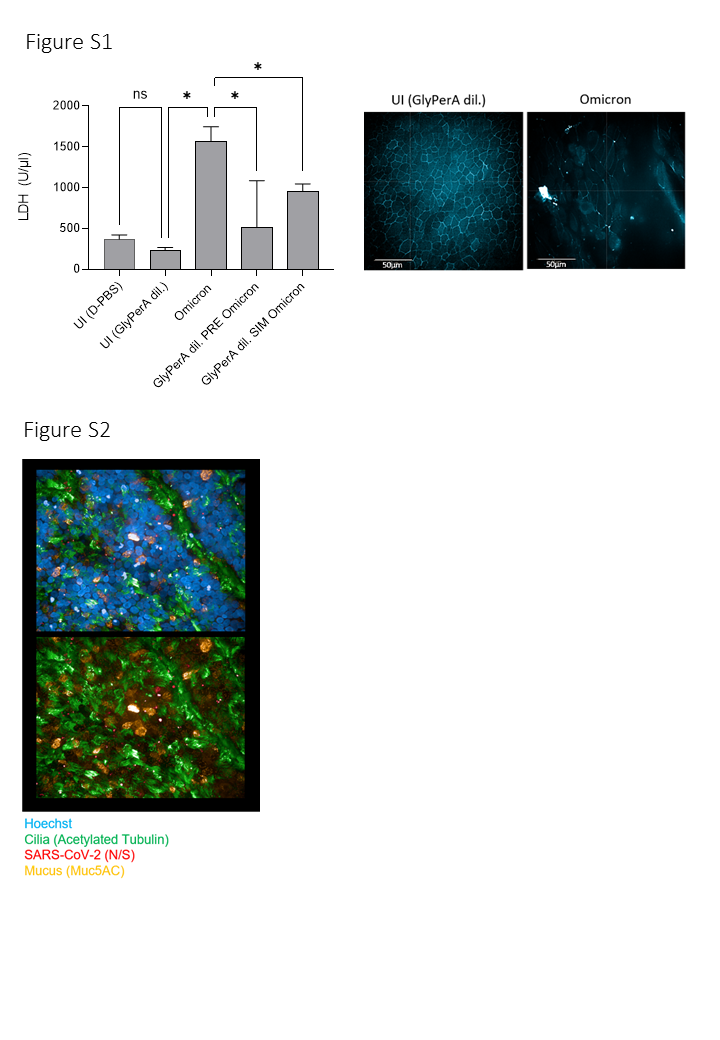

Supplement: Supplementary file 1 — Additional file 1: Fig S1. GlyPerA treatment of 3D human respiratory tissues protects from SARS-CoV-2-mediated cytotoxicity. (left) Cytotoxicity was analyzed using the Cytotoxicity Detection Kit (LDH) from Roche according to the manufacturer´s instructions (Merck, cat# 1164493001, Austria). This kit is based on measuring LDH activity released from damaged cells. Treatment of 3D human respiratory tissue models with GlyPerA at a dilution 1/100 (UI/GlyPerA dil.) did not harm the cells compared to D-PBS-treated control cells. In contrast, SARS-CoV-2 (Omicron)-infected cells showed a significantly higher LDH release, which was significantly down-modulated when cells were either pre-treated with GlyPerA-solution prior infection or when the substance was added simultaneously (GlyPerA dil. PRE Omicron, GlyPerA dil. SIM Omicron). This indicates a significant protection of the tissues from infection by the compound independent on the time of application. *P < 0.05 (right) An occludin staining revealed that GlyPerA-treatment of NHBE respiratory cultures did not have any effect on the tight junctions of epithelia, while in Omicron-infected cultures cells were destroyed. Occludin in blue, scale bars 50 µm. Fig S2. Characterization of ciliated and mucus-producing cells. Ciliated and mucus-producing cells were characterized in NHBE cells at ALI on 1 dpi with SARS-CoV-2 (red). Ciliated cells were stained using acetylated tubulin (green) and goblet cells using MUC5A (orange). The upper picture illustrates all stainings, while in the lower picture the Höchst signal was switched off to see the distribution of ciliated and goblet cells. [file 12931_2023_2397_MOESM1_ESM.tif]
